# On-the-road driving performance the morning after bedtime use of suvorexant 15 and 30 mg in healthy elderly

**DOI:** 10.1007/s00213-016-4375-x

**Published:** 2016-07-16

**Authors:** Annemiek Vermeeren, Eva Vets, Eric F.P.M. Vuurman, Anita C.M. Van Oers, Stefan Jongen, Tine Laethem, Ingeborg Heirman, An Bautmans, John Palcza, Xiadong Li, Matthew D. Troyer, Rebecca Wrishko, Jacqueline McCrea, Hong Sun

**Affiliations:** 1 Department of Neuropsychology and Psychopharmacology, Faculty of Psychology and Neuroscience, Maastricht University, PO Box 616, 6200 MD Maastricht, The Netherlands; 2 SGS Life Science Services, Antwerp, Belgium; 3 Merck Translational Medicine Europe, MSD Europe Inc., Brussels, Belgium; 4 Merck & Co., Inc., Kenilworth, NJ USA

**Keywords:** Hypnotics, Suvorexant, Zopiclone, Orexin antagonist, Driving, Elderly

## Abstract

**Rationale:**

Suvorexant is a first-in-class orexin receptor antagonist for treating insomnia. There is a general concern that hypnotics may impair next-morning driving ability.

**Objective:**

The objective of this study was to evaluate next-morning driving performance in older adults after single and repeated doses of suvorexant.

**Methods:**

Double-blind, randomized, placebo-controlled, 4-period crossover study in 24 healthy volunteers (10 females), aged 65–80 years. Subjects were treated with suvorexant (15 and 30 mg) for eight consecutive nights, zopiclone 7.5 mg nightly on days 1 and 8, and placebo. Driving performance was assessed on days 2 and 9 (9 h after dosing) using a 1-h standardized highway driving test in normal traffic, measuring standard deviation of lateral position (SDLP). Drug-placebo differences in SDLP >2.4 cm were considered to reflect clinically meaningful driving impairment.

**Results:**

Driving performance as measured by SDLP was not impaired following suvorexant. Mean drug–placebo differences in SDLP following suvorexant 15 and 30 mg on day 2 and 9 were 0.6 cm or less. Their 90 % CIs were all below the threshold of 2.4 cm for clinical relevance and included zero, indicating effects were not clinically meaningful or statistically significant. Symmetry analysis showed no significant differences between the number of participants who had SDLP differences >2.4 cm and those who had SDLP differences <−2.4 cm following suvorexant.

**Conclusions:**

There was no clinically meaningful residual effect of suvorexant 15 and 30 mg on next-morning driving (9 h after bedtime dosing) in healthy older adults, as assessed by mean changes in SDLP and by the number of participants on drug versus placebo that exceeded a predetermined threshold for clinically meaningful impairment.

**Electronic supplementary material:**

The online version of this article (doi:10.1007/s00213-016-4375-x) contains supplementary material, which is available to authorized users.

## Introduction

Suvorexant (MK-4305, Belsomra®) is a hypnotic drug with a novel mechanism of action, that is approved in the USA since 2014 for the treatment of adults with insomnia, who have difficulty falling asleep and/or staying asleep (Merck & Co., Inc. 2014). Suvorexant acts as a selective antagonist at orexin-1 and orexin-2 receptors (Cox et al. 2010). Orexin (or hypocretin) is produced by neurons in the lateral hypothalamus and plays a major role in the regulation of sleep and wake state via projections to orexin receptors in wake-active monoaminergic and cholinergic systems (De Lecea and Huerta 2014; Fulcher et al. 2014). Orexin neurons are most active during wakefulness and fall silent during sleep thereby contributing to the maintenance of wakefulness. Loss of orexin neurons results in narcolepsy, a condition characterized by excessive daytime sleepiness and uncontrolled sleep/wake transitions, and may be accompanied by cataplexy (a sudden loss of muscle tone).

Suvorexant is well absorbed following oral administration, reaching maximum concentrations in plasma between 1.5 and 4 h after intake (Sun et al. 2013). Plasma concentration decreases thereafter with a half-life of about 12 h, and steady state is reached after 3 days of dosing. Clinical studies have shown that suvorexant in doses of 10 mg or more significantly improves subjective and objective measures of sleep in healthy volunteers and insomnia patients (Sun et al. 2013; Herring et al. 2012, 2016; Michelson et al. 2014). Phase-3 trials included both elderly and non-elderly adults with insomnia (Michelson et al. 2014; Herring et al. 2016). A 1-year multicenter phase-3 trial showed that suvorexant 40 and 30 mg had sustained effects on subjective total sleep time up to 1 year (Michelson et al. 2014). Furthermore, suvorexant was well tolerated and did not show rebound or withdrawal effects upon discontinuation. The most common adverse events associated with suvorexant are primarily extensions of the drug’s pharmacological activity, i.e., somnolence, fatigue, and dry mouth (Michelson et al. 2014). The recommended dose in the USA for both elderly and non-elderly adults with insomnia is 10 mg, which may be increased to a maximum of 20 mg.

A general concern associated with the use of hypnotics is their potential to impair driving ability the morning after use, due to residual sedative effects (Food and Drug Administration 2015; Dassanayake et al. 2011; Smink et al. 2010; Vermeeren 2004). Results from initial screening of suvorexant’s potential to impair next-day performance using psychomotor tests (e.g., simple reaction time tests and digit symbol substitution tests) suggested that residual effects of suvorexant are minor after bedtime doses up to 40 mg (Sun et al. 2013; Herring et al. 2012). Subsequently, a dedicated driving study in a group of 28 healthy non-elderly adults (age range 21–64 years) was conducted to evaluate effects of single and repeated doses of suvorexant 20 and 40 mg on next-morning performance in an on-the-road driving test (Vermeeren et al. 2015). Results showed that measurable impairment occurred after suvorexant as compared to placebo, but the mean effects on SDLP were less severe than previously found for alcohol in blood concentrations of 0.5 g/L (Louwerens et al. 1987), which is the legal limit for driving in most countries. The effects were therefore not considered to be clinically meaningful. Nonetheless, four subjects requested that a total of five driving tests be stopped before scheduled completion, because they felt too drowsy to continue safely. In addition, analysis of individual changes in driving performance showed that a statistically significant larger number of subjects showed impairment as compared to improvement after single doses of 20 and 40 mg, and after repeated doses of 40 mg. Notably, correlations between changes in driving performance and plasma concentrations of suvorexant were very low (<0.3). It was concluded that the residual effects of suvorexant 20 and 40 mg are on average not clinically meaningful, but there may be some individuals who experience next-day effects (Vermeeren et al. 2015).

A question that remains is whether suvorexant has clinically meaningful residual effects on driving in older adults (i.e., >65 years). The highest prevalence of sleeping problems is found in older people, and most users of hypnotics are elderly (Drake et al. 2003; Glass et al. 2005; Bain 2006). Drug effects may be more pronounced in elderly drivers because of age-related reductions in liver capacity and lean body mass. In addition, the sensitivity of the hypnotic effects may be increased (Woodward 1999). The aim of the present study was therefore to evaluate next-morning driving performance in adults aged 65 to 80 years, after single and repeated doses of suvorexant 15 and 30 mg. The doses studied were those evaluated in phase-3 trials in elderly patients (Michelson et al. 2014; Herring et al. 2016). Zopiclone 7.5 mg was selected as an active control to demonstrate assay sensitivity versus placebo, as has been the case in other studies (Leufkens and Vermeeren 2014; Mets et al. 2011; Ramaekers et al. 2011; Vermeeren et al. 2014, 2015).

## Methods

### Participants

Twenty-four participants (14 males and 10 females) were recruited from a database. Healthy volunteers between 65 and 80 years of age (inclusive) were eligible to enroll if they possessed a valid driver’s license, had driving experience of ≥3000 km/year on average within the last 3 years, body mass index between 18 and 30 kg/m^2^ (inclusive), normal vision (corrected or uncorrected), and a regular sleep pattern (bedtime between 21:00 and 24:00). Participants were required to be in good health, as confirmed by their medical history, physical examination, vital sign measurement, electrocardiogram, and laboratory safety tests (blood chemistry and hematology).

Participants who met any of the following criteria were excluded from the study: history or present evidence of any clinically significant physical, neurological, psychiatric, or sleep disorders, alcoholism or drug abuse; use of medication known to affect driving performance or hepatic drug metabolism; estimated creatinine clearance ≤60 mL/min based on the Cockroft-Gault equation; major surgery, blood donation or participation in any other clinical trial within 4 weeks prior to screening; smoking >6 cigarettes per week; alcohol consumption >3 drinks per day; caffeine consumption >6 servings per day (1 serving each equivalent to 120 mg caffeine). All participants were tested for drug use (amphetamines, benzodiazepines, barbiturates, cannabis, cocaine, methadone, tricyclic antidepressant, and opiates), at pre-study screening and at the start of each test session.

During participation subjects were required to abstain from prescription and non-prescription medication, and grapefruits or grapefruit products. They also had to refrain from smoking and/or consuming caffeine and alcohol from the time of arrival at the site on treatment days until the completion of all tests the next day. In addition, alcoholic drinks, fruit juice, caffeine, and food were not permitted from 48, 12, 10, and 4 h prior to arrival, respectively. Dietary restrictions were to avoid effects which could influence driving (alcohol, caffeine, and nicotine) and/or affect the results of pharmacokinetic testing (juice and food). Furthermore, participants were required not to drive their own vehicles from intake of the first dose until 24 h after the last dose of each treatment period.

Ethical approval was obtained from the Medical Ethics Committee of ZNA Middelheim, Antwerp, Belgium, and all volunteers provided written informed consent prior to enrollment. The study was carried out in compliance with guidelines for Good Clinical Practice.

### Design

The study (Merck protocol 039) was conducted from February to June 2011, according to a randomized, double-blind, placebo and active drug controlled, 4-period crossover design. A four by four Latin Square design balanced for first-order carryover was used. Subjects were randomized to one of four treatment sequences, using validated randomization software. Each treatment period lasted for 8 days, and residual effects were assessed in the mornings of day 2 and 9. Treatments were suvorexant 15 mg, suvorexant 30 mg and placebo on days 1 to 8, and zopiclone 7.5 mg on day 1 and 8 only, with placebo given for the 6 days in between (day 2 to 7). Order of treatment conditions was balanced over subjects. Washout between treatments periods was at least 7 days. Single-dose zopiclone was included as an active control to demonstrate assay sensitivity versus placebo on day 2 and day 9.

### Assessments

#### Highway driving test

Residual effects were assessed using a standardized on-the-road driving test, which measures road tracking performance (O’Hanlon 1984). In this test, subjects operated a specially instrumented vehicle for about 1 h over a 100-km (61-mile) primary highway circuit (road E313) between the Belgian cities of Tongres and Diepenbeek, accompanied by a licensed driving instructor having access to dual controls (brakes and accelerator). The subjects’ task is to drive with a steady lateral position between the delineated boundaries of the slower (right) traffic lane, while maintaining a constant speed of 95 km/h (58 mph). Subjects may deviate from those instructions only to pass a slower vehicle and to leave and re-enter the highway at the turnaround points. During the drive, the vehicle’s speed and lateral distance to the left lane-line are continuously recorded. These signals are digitized at a rate of 4 Hz and stored on an onboard computer disk file for later preprocessing and analysis. The primary outcome variable is standard deviation of lateral position (SDLP in cm), which is a measure of “weaving” or road tracking error. The secondary outcome variable is standard deviation of speed (SDS in km/h), which is a measure of speed control. Performance in this test has repeatedly been found sensitive to residual effects of hypnotics, including zopiclone 7.5 mg (Vermeeren et al. 2015; Leufkens and Vermeeren 2009, 2014; Leufkens et al. 2014; Vermeeren et al. 2014).

#### Word learning test

The Word Learning Test is a verbal memory test for the assessment of immediate and delayed free recall and delayed recognition. In this test, a sequence of 15 monosyllabic nouns is shown on a computer display at a rate of 1 per 2 s. Immediately thereafter the subject is required to verbally recall as many words as possible. The sequence is repeated on four more trials, and the sum of separate trial scores is the Immediate Recall Score. After a delay of 30 min, the subject is again required to recall as many words as possible without prompting. The total number of words correctly recalled is the Delayed Recall Score. Finally, the subject is shown a sequence of 30 words on the computer display, including 15 words from the original set and 15 new words in random order. The subject has to indicate as quickly as possible whether a word originates from the original set or not by pressing corresponding buttons. The number and speed of correct responses are recorded as the Recognition Score and the Recognition Reaction Time (in ms), respectively. Nine parallel lists were used, with a different list for each of the 8 testing days and the training. Lists were balanced across treatments. Performance in this test has repeatedly been found sensitive to residual effects of zopiclone 7.5 mg (Vermeeren et al. 1998a, 2002, 2015; Leufkens and Vermeeren 2009; Leufkens et al. 2009, 2014).

#### Body sway

The ability of subjects to maintain a balanced body posture was evaluated by measures of body sway during quiet stance maintenance using a portable AccuSway^Plus^ force platform (Advanced Mechanical Technology Inc., Watertown, MA). In this test, subjects are instructed to stand as still as possible on the platform in an upright position with bare feet placed parallel at hip width, arms relaxed along the body and facing forward. The system measures ground reacting force and movement in three orthogonal directions, providing the center of foot pressure (CoP) coordinates. These data are used to calculate the extent of movement of the CoP during each recording. Dependent variables are the path length of the CoP (in cm) and the surface area of 95 % confidence ellipse enclosing the CoP (A95 in cm^2^). A95 is the primary measure, as it has shown to be a more sensitive measure of postural stability (Boyle et al. 2009; Norris et al. 2005; Otmani et al. 2012). Each test consisted of six 40-s trial recordings comprising two task conditions. In the first three trials, subjects were instructed to keep their eyes open and fixated on a point 50 cm in front of them at eye level. In the following three trials, subjects were instructed to keep their eyes closed. A95 and CoP scores were averaged over each set of three trials. Foot position is standardized between trials by markings on the platform. Recent studies have shown that postural balance as measured with this test is sensitive to low and moderate doses of alcohol, sleep deprivation, and residual effects of hypnotics (Vermeeren et al. 2015; Jongen et al. 2014, 2015).

#### Digit symbol substitution test

The Digit Symbol Substitution Test (DSST) measures processing speed and working memory. It is a computerized version (Ramaekers et al. 1999) of the original paper and pencil test taken from the Wechsler Adults Intelligence Scale. The subject is shown an encoding scheme consisting of a row of squares at the top of a touch-screen, wherein nine digits are randomly associated with particular symbols. The same symbols are presented in a fixed sequence at the bottom of the screen as a row of separate response buttons. The encoding scheme and the response buttons remain visible while the subject is shown successive presentations of a single digit at the center of the screen. The subject is required to match each digit with a symbol from the encoding list as rapidly as possible by touching the corresponding symbol on the touch-screen. The number of digits correctly encoded within 3 min is the performance measure. Performance in this test has previously been found sensitive to residual effects of zopiclone 7.5 mg (Vermeeren et al. 2015; Leufkens et al. 2009; Mets et al. 2011).

#### Subjective ratings

Before starting the cognitive tests, subjects indicated their subjective feelings using the Bond and Lader’s 16-item mood scale for providing three factor analytically defined summary scores: “alertness,” “contentedness,” and “calmness” (Bond and Lader 1974). The scale for alertness summary scores ranges from 0 to 27, where zero corresponds to most alert. The driving instructors used two visual-analog scales for describing the subject’s driving quality and apparent sedation at the conclusion of the driving test, where zero corresponds to best driving quality and lowest sedation.

#### Pharmacokinetics

Blood samples (7 mL) for suvorexant and zopiclone determinations were collected after driving, at approximately 11 h post dose. Plasma samples were stored and frozen at −20 °C and later analyzed. The analytical methods for the determination of suvorexant were based on a liquid–liquid extraction of drug from human plasma. The drug and internal standard were separated using reverse-phase high-performance liquid chromatography (HPLC) and detected with tandem mass spectrometry (LC-MS/MS), employing a heated nebulizer (HN) interface in the positive ion mode and multiple reaction monitoring (MRM) mode. The lower limit of quantification (LLOQ) for this method was 1 ng/mL with a linear calibration range from 1 to 1000 ng/mL. Samples were assayed by WuXi AppTec Co. (Shanghai, China). The analytical methods for the determination of zopiclone were based on a liquid–liquid extraction of drug from human plasma. The drug and internal standard were separated using reverse-phase HPLC and detected with LC-MS/MS. The LLOQ for this method was 0.30 ng/mL with a linear calibration range from 0.30 to 150 ng/mL. Samples were assayed by PharmaNet Canada (Québec, Canada).

### Procedure

Within 2 weeks before the first treatment period, subjects slept one night in the same facilities as during treatment conditions, to overcome possible sleep disturbances associated with sleeping in an unfamiliar environment. In the evening preceding their habituation night and the following morning, subjects were individually trained to perform all tests, including the driving test.

On day 1 of each treatment period, subjects arrived at the Clinical Pharmacology Unit in Antwerp at approximately 17:00, upon which their eligibility and compliance with study restrictions were verified by questioning, urine screens for drugs of abuse and pregnancy, breath testing for alcohol, and measurement of vital signs. If all the previous requirements were fulfilled, subjects were provided with a standard dinner at 18:30 and then transferred, under surveillance to a hotel in the Belgian city Tongres, nearby the driving area.

A maximum of four subjects were treated on the same night and tested the following day with 5-min difference between their activities. Subjects were fasted for at least 4 h prior to dosing. At 23:15, the first subject was administered drug or placebo with 240 mL water in the presence of an investigator and retired to bed. At 07:15 on day 2, the first subject was awakened and vital signs were measured. Following toilet and dress, subjects were provided a standardized light breakfast without caffeine and transported to the highway. The first driving test started at approximately 08:15, i.e., 9 h after bedtime dosing. After completion of the driving test, subjects were transported to the hotel for further assessments. Approximately 11 h after dosing, a blood sample was taken and vital signs were measured. Subsequently, subjects performed the immediate recall part of the Word Learning test, the DSST, the balance test, and the delayed recall and recognition parts of the Word Learning test, in fixed order. Upon completion of all tests at approximately 11:15, subjects were served a light snack and transported back to the Clinical Pharmacology Unit in Antwerp where they remained under supervision for days 2 to 7 of all treatment periods. On day 8, subjects were again transferred to a hotel in the Belgian city Tongres, nearby the driving area, after which the same procedures as outlined above for days 1 and 2 were followed.

Approximately 14 days after the last treatment period, subjects’ health and well-being were confirmed by questioning them about adverse events and by physical examination and laboratory tests (blood chemistry and hematology).

### Statistical analyses

The primary endpoint was mean SDLP. Secondary endpoints were symmetry analysis of individual changes from placebo in SDLP (see below) and mean word learning and body sway test scores. Mean DSST and subjective rating scores were exploratory endpoints. Sample size was determined based on power calculations to rule out a clinically relevant mean difference in SDLP between suvorexant and placebo, defined as the 90 % confidence interval (CI) for the mean difference in SDLP falling below 2.4 cm (equivalent to a one-sided 95 % upper confidence interval for the difference). A mean increase in SDLP of 2.4 cm as compared to placebo corresponds to the effects previously found for alcohol while subjects drove with average blood alcohol concentrations of 0.5 g/L (Louwerens et al. 1987). Assuming a within-subject variance in SDLP of 3.55 cm^2^ based on a previous study (Ramaekers et al. 2011), and a sample size of 24, the study would have a probability of at least 0.80 that the 90 % CI would fall below 2.4 cm if the true mean difference was as high as 1.0 cm.

All performance parameters were analyzed using a linear mixed effects model for repeated measures with fixed factors for *Treatment* (suvorexant 15 and 30 mg, zopiclone, and placebo, abbreviated as S15, S30, ZOP, and PBO, respectively), *Day* (D2 and D9), *Period* (1 to 4), and a *Treatment by Day* interaction and a random factor for *Subject*.

As a secondary analysis, SDLP was also analyzed using symmetry analysis of individual changes from placebo in SDLP. To perform this symmetry analysis, generalized sign tests were used for each treatment condition and treatment day separately to test whether the number of subjects with an increase in SDLP ≥2.4 cm (reflecting impairment) differed significantly from the number of subjects with a decrease in SDLP of −2.4 cm or more (reflecting improvement).

All statistical analyses were done by using the SAS statistical program version 9.3 (SAS Institute Inc. Cary, NC). No adjustments were made for multiple comparisons.

## Results

### Demographic data

All 24 enrolled participants completed the study. Their mean ± standard deviation (SD) age was 68.8 ± 2.7 years (range 65 to 76 years). Their mean ± SD body mass index was 25.8 ± 2.1 kg/m^2^. Mean ± SD body weight was 79.3 ± 7.2 kg for the males and 63.5 ± 9.7 kg for the females. All participants were Caucasian.

### Driving

One driving test, of a 72-year-old female subject, was terminated prematurely by the driving instructor, because he judged the subject too drowsy to continue safely. The test was stopped after 45 min on D2 of PBO. SDLP score for this test was 22.2 cm as calculated from the data collected until the termination of the ride. None of the tests were stopped in S15, S30, and ZOP.

As shown in Table 1, mean changes from placebo in SDLP scores in S15 and S30 were very small on both test days: they ranged from −0.43 (on D2 of S15) to +0.60 (on D9 of S30). None of these changes were statistically significant, or clinically meaningful, as determined by the lower limits of the 90 % CI of these changes which all fell below 0 cm, and the upper limits of the 90 % CI which all fell below the criterion of 2.4 cm, respectively.

**Table 1:** Model-based mean performance scores and mean drug-placebo changes (90 % CI) at both test days in each treatment condition, *n* = 24

		Means							Treatment differences vs PBO, mean (90 % CI)					
	Day	PBO		S15		S30		ZOP	S15		S30		ZOP	
Driving test														
SDLP (cm)	2	16.67	16.24		17.04		18.56		−0.43	(−1.10,0.23)	0.37	(−0.30,1.03)	*1.89*	*(1.22,2.55)*
	9	15.41	15.50		16.01		16.58		0.09	(−0.58,0.76)	0.60	(−0.06,1.27)	*1.17*	*(0.51,1.84)*
SDS (km/h)	2	2.00	2.06		2.11		2.18		0.07	(−0.05,0.18)	0.11	(−0.01,0.23)	*0.18*	*(0.06,0.30)*
	9	1.90	1.98		2.00		2.06		0.08	(−0.04,0.20)	0.11	(−0.01,0.22)	*0.16*	*(0.05,0.28)*
Word learning test														
Immediate recall (number correct)	2	43.7	45.4		45.3		43.6		1.7	(−0.9, 4.3)	1.6	(−1.0, 4.2)	−0.0	(−2.7, 2.6)
	9	45.5	45.7		46.6		44.9		0.2	(−2.4, 2.8)	1.1	(−1.5, 3.7)	−0.6	(−3.2, 2.0)
Delayed recall (number correct)	2	8.6	8.8		8.6		7.8		0.3	(−0.7, 1.2)	0.0	(−0.9, 1.0)	−0.8	(−1.7, 0.2)
	9	8.9	9.2		8.8		8.0		0.3	(−0.7, 1.3)	−0.0	(−1.0, 0.9)	−0.8	(−1.8, 0.1)
Recognition (number correct)	2	26.8	27.3		27.2		26.8		0.5	(−0.4, 1.4)	0.3	(−0.5, 1.2)	−0.1	(−1.0, 0.8)
	9	26.9	27.1		26.6		25.8		0.3	(−0.6, 1.1)	−0.3	(−1.2, 0.6)	*−1.0*	*(−1.9, −0.2)*
Recognition speed (ms)	2	834	822		827		845		−12	(−47, 22)	−8	(−43, 27)	11	(−24, 46)
	9	821	831		805		847		10	(−25, 45)	−16	(−51, 19)	26	(−9, 61)
Body Sway Test														
A95 eyes open^a^ (cm^2^)	2	1.15	1.18		1.28		1.25		1.03	(0.89, 1.18)	1.11	(0.97, 1.28)	1.08	(0.94, 1.25)
	9	1.16	1.08		1.19		1.26		0.93	(0.81, 1.08)	1.03	(0.89, 1.18)	1.09	(0.94, 1.25)
A95 eyes closed^a^ (cm^2^)	2	1.74	1.63		1.85		1.93		0.94	(0.83, 1.06)	1.07	(0.95, 1.20)	1.11	(0.98, 1.25)
	9	1.76	1.73		1.52		1.89		0.98	(0.87, 1.11)	0.87	(0.77, 0.98)	1.08	(0.95, 1.21)
CoP eyes open^a^ (cm)	2	50.7	52.0		51.5		50.0		1.03	(0.98, 1.07)	1.02	(0.98, 1.06)	0.99	(0.95, 1.03)
	9	50.3	49.4		50.9		48.0		0.98	(0.94, 1.02)	1.01	(0.97, 1.05)	*0.95*	*(0.92, 0.99)*
CoP eyes closed^a^ (cm)	2	66.4	66.8		67.1		63.7		1.01	(0.96, 1.06)	1.01	(0.96, 1.06)	0.96	(0.91, 1.01)
	9	64.3	63.3		62.3		62.4		0.98	(0.94, 1.03)	0.97	(0.92, 1.02)	0.97	(0.92, 1.02)
Digit Symbol Substitution Test														
DSST (number correct)	2	71.1	71.8		72.3		71.5		0.8	(−2.7, 4.2)	1.2	(−2.3, 4.6)	0.5	(−3.0, 3.9)
	9	74.0	72.7		74.9		73.2		−1.3	(−4.8, 2.2)	0.9	(−2.6, 4.4)	−0.8	(−4.3, 2.6)
Subjective rating scales														
Instructor-rated Driving quality (mm)	2	33.4	35.9		37.2		38.0		2.5	(−1.7, 6.6)	3.8	(−0.4, 7.9)	*4.6*	*(0.5, 8.8)*
*Very well (0)–Very bad (100)*	9	35.3	35.5		33.7		38.9		0.2	(−3.9, 4.4)	−1.5	(−5.7, 2.6)	3.6	(−0.5, 7.8)
Instructor-rated sedation (mm)	2	14.8	12.5		7.5		12.4		−2.3	(−6.8, 2.3)	*−7.3*	*(−11.8, −2.8)*	−2.4	(−6.9, 2.1)
*None (0)–Completely (100*)	9	6.9	8.1		9.8		9.5		1.3	(−3.3, 5.8)	3.0	(−1.6, 7.5)	2.6	(−1.9, 7.1)
Subject-rated alertness (mm)	2	17.2	17.3		17.2		16.9		0.1	(−0.9, 1.1)	0.0	(−1.0, 1.0)	−0.3	(−1.2, 0.7)
*Very alert (0)–Not alert (27)*	9	15.8	15.6		16.1		15.3		−0.2	(−1.2, 0.8)	0.3	(−0.7, 1.3)	−0.5	(−1.5, 0.5)

^a^Changes reflect fold-changes from placebo. Significant differences (values with CIs that do not overlap zero or 1 in the case of fold-change values) are in italics. PBO, placebo; S15, suvorexant 15 mg; S30, suvorexant 30 mg; ZOP, zopiclone 7.5 mg

In ZOP, mean SDLP was increased by 1.89 cm (90 % CI 1.22 to 2.55) on D2 and by 1.17 cm (90 % CI 0.51 to 1.84) on D9. These results show that effects of zopiclone on driving were statistically significant on both days (demonstrating assay sensitivity) and clinically relevant on D2, but not on D9.

**Fig. 1:**
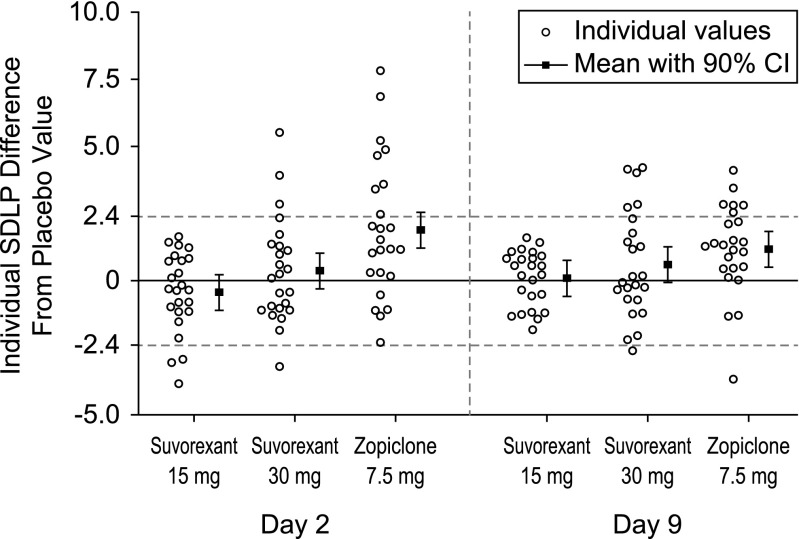
Individual SDLP (cm) differences from placebo (mean and 90 % confidence interval) by treatment and day following bedtime administration of suvorexant (MK-4305) 15 mg, 30 mg single dose (day 2) and multiple doses (day 9), and single dose of zopiclone 7.5 mg (day 2 and day 9), *n* = 24

Individual changes from placebo in SDLP are shown in Fig. 1.

Symmetry analysis showed that significantly more subjects showed an increase in SDLP ≥2.4 cm than a decrease in SDLP of the same magnitude on both treatment days in ZOP, but not in S15 and S30 (Table 2).

**Table 2:** Symmetry analysis of numbers of subjects whose SDLP increased more than 2.4 cm (indicating impairment) and numbers of subjects whose SDLP decreased more than 2.4 cm (indicating improvement), *n* = 24

Treatment condition	Day	∆SDLP ≥2.4 cmn (proportion)	∆SDLP ≤−2.4 cmn (proportion)	Test statistic^a^	Reject H_0_
S15	2	0 (0.00)	3 (0.13)	−1.73	No
	9	0 (0.00)	0 (0.00)	ND	No
S30	2	3 (0.13)	1 (0.04)	1	No
	9	5 (0.21)	1 (0.04)	1.63	No
ZOP	2	8 (0.33)	0 (0.00)	2.83	Yes
	9	6 (0.25)	1 (0.04)	1.89	Yes

^a^General sign test. Reject H_0_ (null hypothesis) if statistic >1.74 (indicates statistically significant imbalance). S15, suvorexant 15 mg; S30, suvorexant 30 mg; ZOP, zopiclone 7.5 mg

In line with results of SDLP, variability in speed (SD Speed) was significantly increased compared to placebo on D2 and D9 in ZOP, but not in S15 and S30 (Table 1).

### Word learning

No statistically significant impairment was observed in immediate and delayed recall of words and in speed of recognition in the Word Learning Test (Table 1). Only the total number of words correctly recognized showed significant impairment in ZOP on D9.

### Body sway

No statistically significant impairment in body sway was observed (Table 1).

### DSST

No statistically significant impairment in DSST performance was observed (Table 1).

### Subjective evaluations

Subjects did not judge themselves significantly less alert in any treatment condition as compared to placebo. Similarly, the driving instructors did not judge the subjects as appearing significantly more sedated in any treatment condition as compared to placebo. Yet, they did rate the subjects driving quality as significantly worse than placebo on D2 in ZOP (Table 1).

### Blood samples

On D2 mean (range) plasma concentrations of suvorexant (C11h) were 0.259 (0.147 to 0.394) and 0.399 (0.217 to 0.687) μM, for 15 and 30 mg doses, respectively. On D9 mean C11h increased to 0.394 (0.198 to 0.664) and 0.640 (0.312 to 1.241) μM, respectively. Mean (range) plasma concentrations (C11h) of zopiclone were 19.19 (8.15 to 33.47) ng/ml on D2 and 17.97 (7.61 to 28.35) ng/ml on D9. There appeared to be no clear relation between plasma concentrations of suvorexant (pooled over dose) and SDLP, as shown by plots of individual SDLP difference from placebo scores versus suvorexant plasma concentration by gender (Supplementary Fig. S1).

### Safety

Table 3 presents a summary of adverse events (AEs) reported by ≥5 % of the subjects after any treatment. After suvorexant, the most common AEs were somnolence (8.3 % with S15; 29.2 % with S30), headache (8.3 % with S15; 20.8 % with S30), and poor sleep quality (25.0 % with S15; 0.0 % with S30).

**Table 3:** Summary of AEs reported by at least 5 % of the subjects after any treatment, *n* = 24

	PBO^a^ *n* (%)	S15 *n* (%)	S30 *n* (%)	ZOP *n* (%)
Subjects with at least 1 AE	13 (54.2)	14 (58.3)	13 (54.2)	11 (45.8)
Headache	7 (29.2)	2 (8.3)	5 (20.8)	3 (12.5)
Somnolence	4 (16.7)	2 (8.3)	7 (29.2)	3 (12.5)
Dysgeusia	0 (0.0)	0 (0.0)	0 (0.0)	3 (12.5)
Fatigue	0 (0.0)	2 (8.3)	1 (4.2)	1 (4.2)
Dizziness	0 (0.0)	0 (0.0)	0 (0.0)	2 (8.3)
Poor sleep quality	3 (12.5)	6 (25.0)	0 (0.0)	0 (0.0)
Nightmare	3 (12.5)	1 (4.2)	2 (8.3)	1 (4.2)

^a^Includes placebo treatment days 2 to 7 in ZOP. PBO, placebo; S15, suvorexant 15 mg; S30, suvorexant 30 mg; ZOP, zopiclone 7.5 mg

After zopiclone, somnolence, headache, and dysgeusia were the most common AEs (all 12.5 %). The remaining AEs were reported by no more than two (8.3 %) subjects.

After placebo (which includes placebo treatment days 2 to 7 in ZOP), 13 subjects reported at least one adverse experience. All AEs were rated as mild or moderate in intensity. Headache was the most frequently reported AE after placebo (29.2 %), followed by somnolence (16.7 %), poor sleep quality (12.5 %), and nightmares (12.5 %). One female subject was reported to have severe somnolence on D2 of PBO which resulted in premature termination of the driving test.

No serious AEs and events of clinical interest, such as cataplexy, were reported in this study, and there were no consistent treatment-related changes in laboratory, vital signs, or ECG safety parameters.

## Discussion

The primary aim of the current study was to assess the residual effects of suvorexant 15 and 30 mg on car driving in older adults, 9 h after single and repeated bedtime dosing. The primary measure of driving performance was SDLP. Comparison of mean drug-placebo changes and 90 % CIs to a pre-defined criterion of 2.4 cm showed no clinically meaningful or statistically significant effects of single and repeated doses of suvorexant 15 and 30 mg on SDLP. All mean changes were 0.6 cm or less, and the 90 % CIs of these changes lay well below the criterion of 2.4 cm and included zero. Symmetry analyses of the numbers of subjects showing drug–placebo changes in SDLP exceeding 2.4 or −2.4 cm showed a similar lack of treatment effect. In line with this lack of effects on SDLP, no significant effects of suvorexant on SD of speed were observed.

Assay sensitivity was demonstrated by zopiclone 7.5 mg on both days. Following this drug SDLP and SD speed significantly increased as compared to placebo on D2 and D9. On D2, the mean change in SDLP was +1.9 cm and 90 % CI included 2.4 cm, indicating that impairment was also clinically meaningful. On D9, SDLP increased by 1.2 cm, and the 90 % CI lay above zero, but below 2.4 cm, indicating that the effect was statistically significant (demonstrating assay sensitivity), but not clinically meaningful.

Secondary and exploratory aims were to assess suvorexant’s residual effects on memory, digit symbol substitution, and postural balance. Results showed no significant differences between suvorexant 15 and 30 mg and placebo on performance in any of these tests. However, also no effects of zopiclone were observed on immediate and delayed recall, DSST and balance. Only word recognition was impaired by zopiclone on D9.

Subjective ratings of driving quality were in line with the performance effects as measured by SDLP. The driving instructors rated the subjects’ driving quality as significantly worse than placebo only after the first dose of zopiclone. After suvorexant and the second dose of zopiclone driving quality was not judged to differ from placebo. Contrary to ratings of driving quality, subjective ratings of sedation did not discriminate between treatments.

In summary, there were no clinically meaningful or statistically significant differences between effects of suvorexant 15 and 30 mg and placebo in a group of elderly volunteers, in driving, cognitive and psychomotor performance, and subjective evaluations of performance and sedation. The lack of residual effects in elderly subjects generally supports the conclusions of a previous study in non-elderly (21–65 years) volunteers, also showing no clinically meaningful residual effects of suvorexant (20 and 40 mg) with regard to mean changes in SDLP (Vermeeren et al. 2015). However, the results of the non-elderly study suggested that there may be some individuals who experience next-day effects, as suggested by individual changes in SDLP and prematurely stopped tests. In the present study, after the 15 mg dose, which is in the FDA-approved dose range, none of the elderly subjects showed driving impairment on either day 2 or day 9. On the contrary, three subjects driving seemed to improve after this dose on D2 (see Fig. 1).

The mean change in SDLP after the first dose of zopiclone on SDLP was +1.9 cm, and the associated 90 % CI included the criterion of 2.4 cm. This shows that the zopiclone effect was comparable to that of driving under the influence of alcohol with a blood alcohol concentration of 0.5 g/L, which is the legal limit for driving in most countries. The effect of zopiclone on D2 in the present study is comparable to that found previously in healthy older drivers (Leufkens and Vermeeren 2009). The effect was smaller on D9 (+1.2 cm). A similar decrease in the effect of zopiclone from D2 to D9 with repeat of single doses was found in a previous study with suvorexant in non-elderly subjects (Vermeeren et al. 2015). Although the difference in effects on D2 and D9 is small, it does raise the question of what may have caused it. Since zopiclone was administered as single doses, these findings cannot easily be explained by development of physiological tolerance. Plasma concentrations of zopiclone were comparable on both days. Practice effects are also not likely. Driving performance after placebo does not consistently improve with repeated testing. Out of 14 studies repeatedly assessing the driving performance after placebo, six showed a decrease in mean SDLP with repeated testing, whereas eight studies showed an increase in mean SDLP (Vermeeren and O’Hanlon 1998; Vermeeren et al. 2002, 2015; Ramaekers et al. 1994, 1995, 2006, 2011; Brookhuis et al. 1993; Conen et al. 2011; O’Hanlon et al. 1998; Robbe and O’Hanlon 1995; Theunissen et al. 2006; Verster et al. 2003; Wingen et al. 2005). In the present study, SDLP decreased slightly from day 2 to day 9 after placebo. This improvement is smaller however, than the reduction in effect of zopiclone. As suggested for the previous study (Vermeeren et al. 2015), the reduced effect of zopiclone could be due to the development of behavioral tolerance after single exposure to its effects, i.e., development of behavioral strategies to compensate for the drug’s effects on driving performance, as seen with alcohol (Vogel-Sprott 1997).

The lack of residual effects of zopiclone in word learning, DSST, and body sway is in contrast with results of previous studies showing significant residual impairment after zopiclone in these tests (Vermeeren et al. 1998, 2002, 2015; Leufkens and Vermeeren 2009; Leufkens et al. 2009; Mets et al. 2011). The discrepancy may be due to age-related differences in placebo performance between studies. Subjects in the present study were older than in previous studies, and their performance in these tests was relatively worse. It may be that their placebo performance in these tests did not allow much further impairment due to zopiclone (i.e., floor effects).

On average subjects’ SDLP scores in the placebo condition were comparable to that in studies with younger volunteers (Vermeeren et al. 2014, 2015; Jongen et al. 2015). Only one driving test, for a 72-year-old female subject, was terminated prematurely by the driving instructor after 45 min on D2 in period 4, while on placebo treatment. The driving instructor judged the subject too drowsy to continue safely. No driving tests were stopped after subjects received suvorexant or zopiclone in this study. Termination of driving tests after placebo has occurred before, although the prevalence is low. A review of 47 published papers on double-blind, placebo-controlled studies using the highway driving test to assess drug effects on driving, found that 0.7 % of the driving tests had been terminated before completion after placebo treatment, whereas 4.1 % were stopped after drug treatment. The results confirmed the conclusion that the decision to stop a driving test is not a reliable correlate of objective driving performance (O’Hanlon 1984; Verster and Roth 2012).

Plasma concentrations of suvorexant were in the expected dose-range, and in line with those found in the previous study assessing effects of suvorexant 20 and 40 mg on driving in non-elderly subjects. Plasma concentrations of zopiclone in this sample of elderly (19.2 and 18.0 ng/ml) were on average slightly increased compared to those previously found in non-elderly (15.6 and 15.8 ng/ml).

The use of healthy volunteers instead of patients could be considered a limitation of this study. An important reason to study the effects of suvorexant in healthy volunteers is to facilitate comparisons to previous driving studies, which were virtually all conducted with normal volunteers (Vermeeren 2004, Vermeeren et al. 2014; Leufkens et al. 2009, 2014; Mets et al. 2011; Ramaekers et al. 2011). More importantly, a recent study comparing the effects of zopiclone on driving in middle-aged patients with insomnia and middle-aged healthy volunteers suggests that healthy volunteers may be more sensitive to the residual effects (Leufkens et al. 2014). Thus, studying drug effects in healthy volunteers minimizes the risk of failing to detect clinically relevant impairment associated with use of a drug.

In conclusion, results of the present study show that single and repeated doses of suvorexant 15 and 30 mg are not associated with next-day residual impairment in elderly subjects as assessed by on-the-road driving performance, balance, and cognitive tests. Due to individual variation, patients taking suvorexant should be advised not to drive, operate machinery, or engage in other activities requiring full mental alertness until fully awake.

## Electronic supplementary material

Supplementary Figure S1 Individual SDLP differences from placebo versus suvorexant plasma concentrations by gender at day 2 (top) and day 9 (bottom) 113 × 155mm (300 × 300 DPI) (JPEG 68 kb)

High Resolution Image (DOC 231 kb)

## Abbreviations

A95: Area of 95 %
AE: Adverse event
BAC: Blood alcohol concentration
CI: Confidence interval
CoP: Center of pressure
D2: Day 2
D9: Day 9
DORA: Dual orexin receptor antagonist
DSST: Digit symbol substitution test
GABA: Gamma amino butyric acid
h: Hour(s)
HPLC: High-performance liquid chromatography
km: Kilometers
LC-MS/MS: Tandem mass spectrometry
LLOQ: Lower limit of quantification
PBO: Placebo
S15: Suvorexant 15 mg
S30: Suvorexant 30 mg
SD: Standard deviation
SDLP: Standard deviation of lateral position
SDS: Standard deviation speed
ZOP: Zopiclone 7.5 mg
