# Supplementary figures and images for: On-the-road driving performance the morning after bedtime use of suvorexant 15 and 30 mg in healthy elderly
Source: Psychopharmacology (Berl). 2016 Jul 16;233:3341–51. doi: 10.1007/s00213-016-4375-x (PMC4989000; doi:10.1007/s00213-016-4375-x)

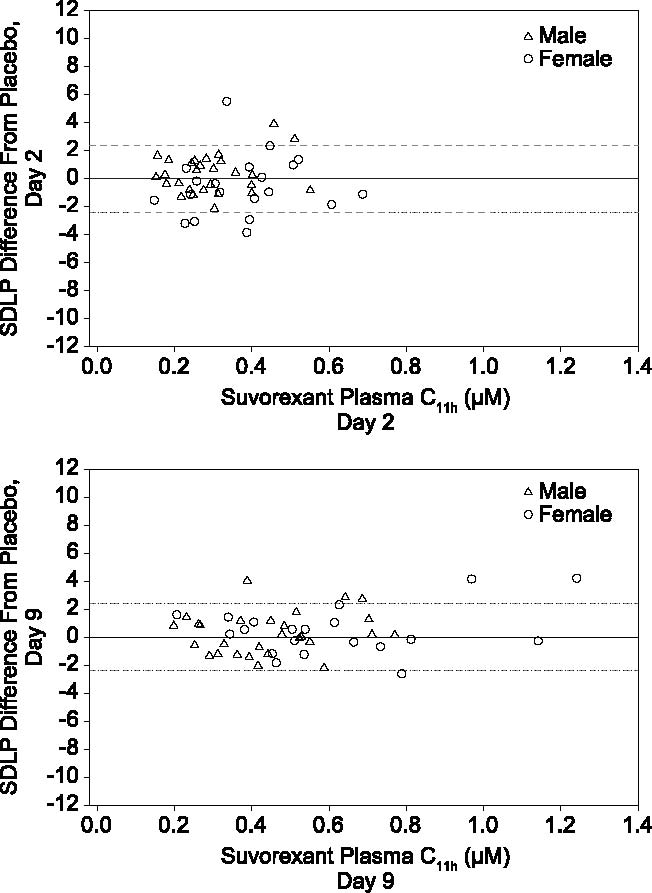

Supplement: Supplementary file 1 — Individual SDLP differences from placebo versus suvorexant plasma concentrations by gender at day 2 (top) and day 9 (bottom) 113 × 155mm (300 × 300 DPI) (JPEG 68 kb) [file 213_2016_4375_Fig2_ESM.jpg]
