# Supplementary material for: On-the-road driving performance the morning after bedtime use of suvorexant 15 and 30 mg in healthy elderly
Source: Psychopharmacology (Berl). 2016 Jul 16;233:3341–51. doi: 10.1007/s00213-016-4375-x (PMC4989000; doi:10.1007/s00213-016-4375-x)
Supplement: Supplementary file 2 — High Resolution Image (DOC 231 kb) [file 213_2016_4375_MOESM1_ESM.doc]

**Supplementary Fig S1** Individual SDLP differences from placebo versus suvorexant plasma concentrations by gender at Day 2 (top) and Day 9 (bottom)

**
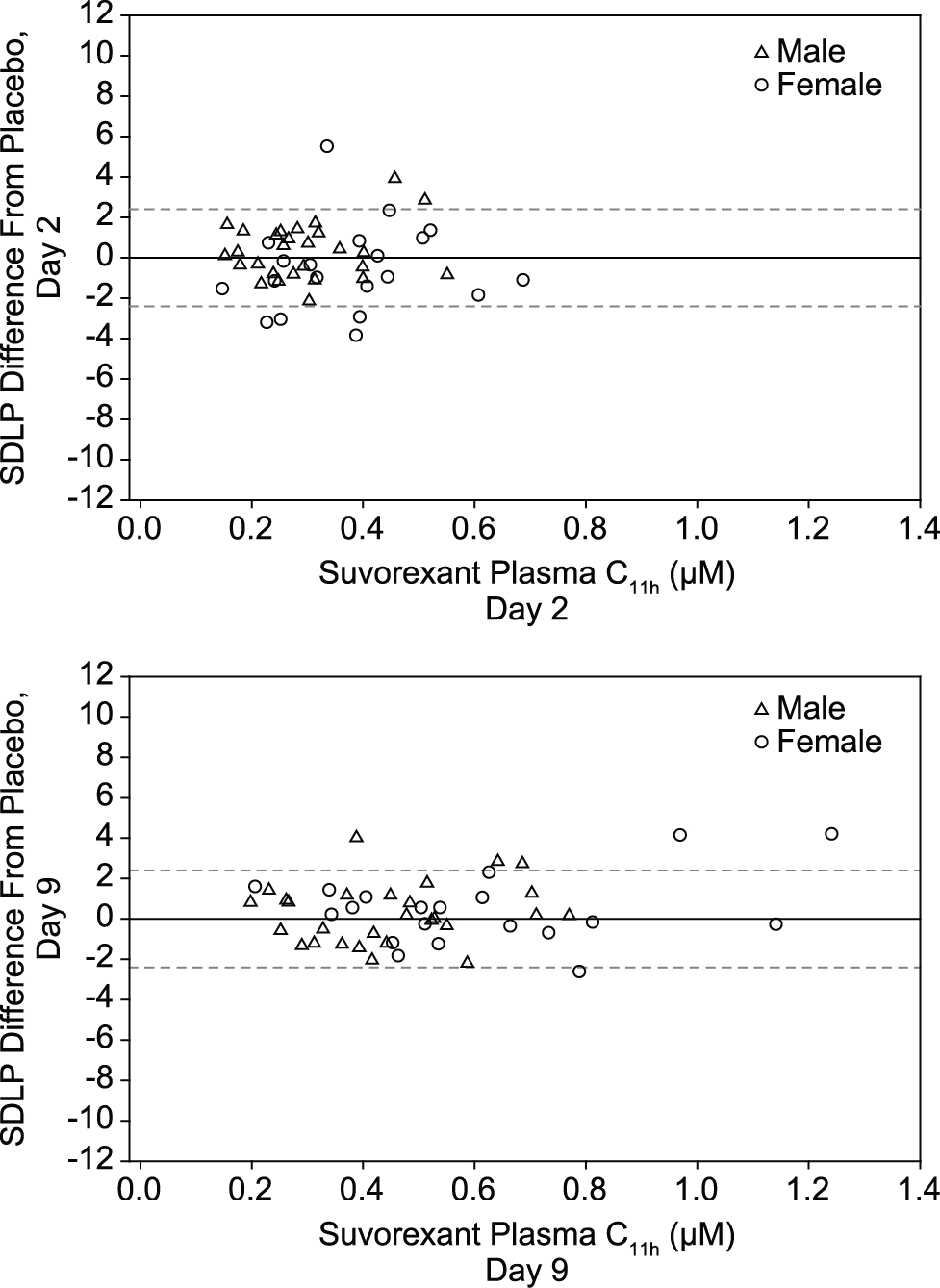
**
